# Identifying Clinical and Genomic Features Associated With Chronic Kidney Disease

**DOI:** 10.3389/fdata.2020.528828

**Published:** 2021-01-14

**Authors:** M. Megan Moreno, Travaughn C. Bain, Melissa S. Moreno, Katherine C. Carroll, Emily R. Cunningham, Zoe Ashton, Roby Poteau, Ersoy Subasi, Michael Lipkowitz, Munevver Mine Subasi

**Affiliations:** ^1^ Department of Mathematical Sciences, Florida Institute of Technology, Melbourne, FL, United States; ^2^ Department of Biomedical and Chemical Engineering and Sciences, Melbourne, FL, United States; ^3^ Department of Biology, University of Florida, Gainesville, FL, United States; ^4^ Department of Mathematics, SUNY Potsdam, Potsdam, NY, United States; ^5^ Department of Computer Engineering and Sciences, Florida Institute of Technology, Melbourne, FL, United States; ^6^ Department of Medicine, Georgetown University Medical Center, Washington, DC, United States

**Keywords:** classification, genomic analysis, AASK, chronic kidney disease, decision trees

## Abstract

We apply a pattern-based classification method to identify clinical and genomic features associated with the progression of Chronic Kidney disease (CKD). We analyze the African-American Study of Chronic Kidney disease with Hypertension dataset and construct a decision-tree classification model, consisting 15 combinatorial patterns of clinical features and single nucleotide polymorphisms (SNPs), seven of which are associated with slow progression and eight with rapid progression of renal disease among African-American Study of Chronic Kidney patients. We identify four clinical features and two SNPs that can accurately predict CKD progression. Clinical and genomic features identified in our experiments may be used in a future study to develop new therapeutic interventions for CKD patients.

## 1 Introduction

The main function of kidney is to remove excess water and waste products from blood. It also helps to regulate the levels of minerals such as sodium, calcium, and potassium in blood. One suffers from chronic kidney disease (CKD), also known as renal disease, when kidney losses its function gradually and usually permanently. CKD, defined by reduced glomerular filtration rate (GFR), proteinuria, or structural kidney disease, is a worldwide growing public health problem^1^. Many subjects with renal disease of most etiologies progress to severe renal failure and/or end stage renal disease (ESRD), requiring renal replacement therapy, which may involve a form of dialysis or renal transplantation (Lewis et al., 1993; Klahr et al., 1994; DCCT, 1995; Brenner et al., 2001; Lewis et al., 2001; Wright et al., 2002; Niki et al., 2015). However, progression rate of CKD is very heterogeneous (Lindeman et al., 1985; Lindeman, 1990; Hallan et al., 2006). While a few predictive factors for progression such as proteinuria have been detected, identification of those at risk to progress remains a significant problem. It has also been established that there are several therapies that can ameliorate the progression of renal disease including ACE inhibitors, blood pressure control, tight diabetes control and perhaps low protein diets; however, in trials examining these therapeutic modalities there remains a very significant risk of progression of renal disease in the subjects receiving optimal therapy (Lewis et al., 1993; Klahr et al., 1994; DCCT, 1995; Brenner et al., 2001; Lewis et al., 2001; Wright et al., 2002; Niki et al., 2015).

African-American Study of Chronic Kidney disease with Hypertension (AASK) was motivated by the high rate of hypertension-related chronic kidney disease in the African-American population and the scarcity of effective therapies. The study involved 21-center randomized double-blinded treatment trial of 1,094 African-American patients with hypertension at ages ranging from 18 to 70 years. Patients had renal failure with GFR between 20 and 65 ml/min/1.73m^2^. Patients were randomized to the angiotensinogen converting enzyme inhibitor (ACEi) ramipril, the β-blocker (BB) metoprolol or the dihydropyridine calcium channel blocker (CCB) amlodipine, and to usual (mean arterial pressure (MAP 102–107) or low (MAP <92) blood pressure (BP) goals. The rationale for the treatment arms was that there was human and animal data suggesting that ACEi and CCB might slow progression of renal disease independent of their BP effects (Lewis et al., 1993; Hallan, 1998), and there was data from observational and treatment studies that a lower BP might have beneficial effects (Klahr et al., 1994; Klag et al., 1997). Although other studies had attempted to achieve a 10 mmHg MAP separation (Hansson et al., 1998; Lewis et al., 2001), AASK is the first major trial to actually achieve this goal. The primary outcome was rate of decline of GFR (GFR slope) based on iothalamate GFR studies at 6 months intervals, with a secondary clinical composite outcome of end stage renal disease (ESRD), a 25 ml/min or 50% drop in GFR from baseline (GFR event), or death (Subasi et al., 2017).

The initial AASK results were not conclusive (Wright et al., 2002). While the adopted therapy was shown to slow the progression of renal disease, there was still high rate of progression to renal failure. The CCB arm of the study was stopped early when interim analysis indicated that CCB was inferior to both BB and ACEi in patients with >0.22 urine protein/creatinine ratio (about 300 mg proteinuria/24 h) (Agodoa et al., 2001). The low BP goal of the study did not improve outcomes: there was no beneficial effect of low MAP on rate of progression of renal disease as defined by GFR slope or clinical composite outcomes (GFR events, end stage renal disease (ESRD) or death). Subsequently, a similar result was found in the REIN trial (Ruggenenti et al., 1999). Studies in Type 2 diabetes have demonstrated a linear relation of achieved BP to renal outcomes (Bakris et al., 2003; Pohl et al., 2005); however, it should be noted that all the patients in these studies were treated to the same goal BP, so that rather than low BP being protective, the ability to achieve lower BPs may have defined a sub-population in these studies with low risks of disease progression. Despite the lack of effect on renal outcomes in AASK, proteinuria was diminished by the lower BP goal. This finding is similar to that previously reported for diabetics (Lewis et al., 2001). Finally, a subgroup analysis in AASK did suggest that patients on a non-protective regimen (CCB) may have benefited from the low BP goal (Contreras et al., 2005). Most importantly in AASK, ACEi decreased the number of events as compared to both BB and CCB (Wright et al., 2002). These data for ACEi vs. CCB are tabulated in Table 1 (risk reduction adjusted for baseline covariates) and were most dramatic for the hard outcomes, especially ESRD.

**TABLE 1:** Analysis of clinical composite outcomes - 95% confidence interval (CI).

ramipril vs. Amlodipine	% Risk Reduction	95% CI	*p*-value
GFR event, ESRD or death	38%	14%−56%	0.004
GFR event or ESRD	40%	14%−59%	0.006
ESRD or death	49%	26%−65%	<0.001
ESRD alone	59%	36%−74%	<0.001

Several possible interventions such as blood pressure control (Wright et al., 2002), diabetes treatment (DCCT, 1995), controlling dietary protein intake (Klahr et al., 1994) and medications with possible renoprotective effects (Ruggenenti et al., 1999; Agodoa et al., 2001; Wright et al., 2002) have been tested in clinical trials. In all cases, the residual rate of progression of chronic kidney disease has remained significant. To date, there are few prediction models to identify which patients are likely to progress significantly. Subasi et al. (2017) (Subasi et al., 2017) identified serum proteomic patterns that can accurately distinguish rapid progression and slow progression among AASK patients. Recently, Lipkowitz et al. (2013) (Parsa et al., 2013) examined effects of variants in gene encoding apolipoprotein L1 (APOL1) on the disease progression and observed that renal risk variants in APOL1 were associated with the higher rates of ESRD and progression of chronic kidney disease in African-American patients as compared to white patients. Other recent studies include Rahman et al. (2013), where the effects of two antihypertensive drug dose (PM dose and add-on dose) schedules on nocturnal blood pressure vs. usual therapy (AM dose) in former participants were determined and Chen et al. (2016), where the longitudinal changes in hematocrit in hypertensive renal disease were studied.

The goal of our current study is to apply a pattern-based classification method to identify clinical and genomic features that may serve as prognostic markers for the progression of renal disease among AASK patients. Clinical and genomic features identified in our analysis shall be used in a future study to obtain comparison of the disease progression in white patients and African-American patients, both of those with and those without apolipoprotein L1 (APOL1) high-risk variants. The ultimate goal of our AASK data analysis, started in (Subasi et al., 2017) and continued in this current work, is to identify new targets and provide basis for new therapeutic interventions for chronic kidney disease.

## 2 Study Subjects

**FIGURE 1:**
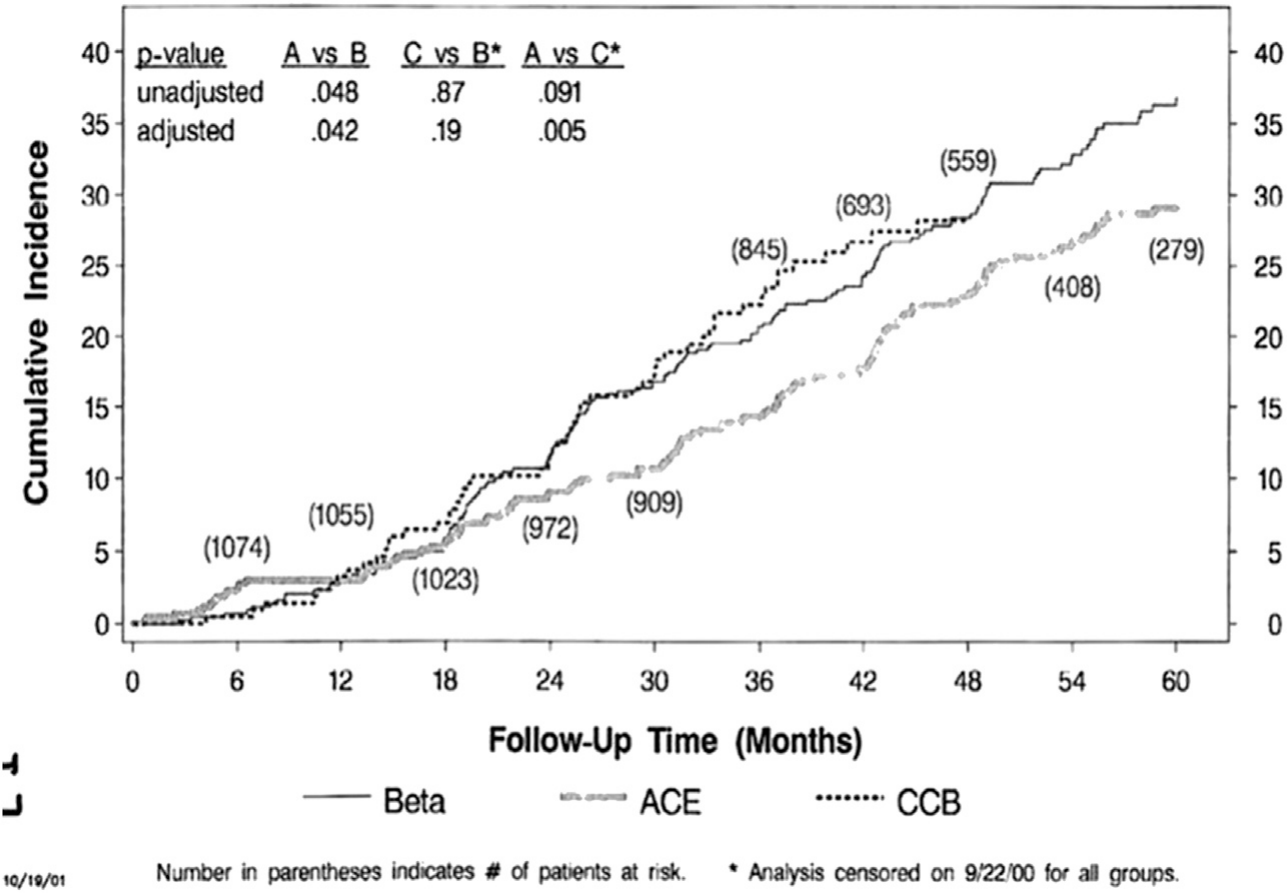
AASK clinical composite events–all patients.

**FIGURE 2:**
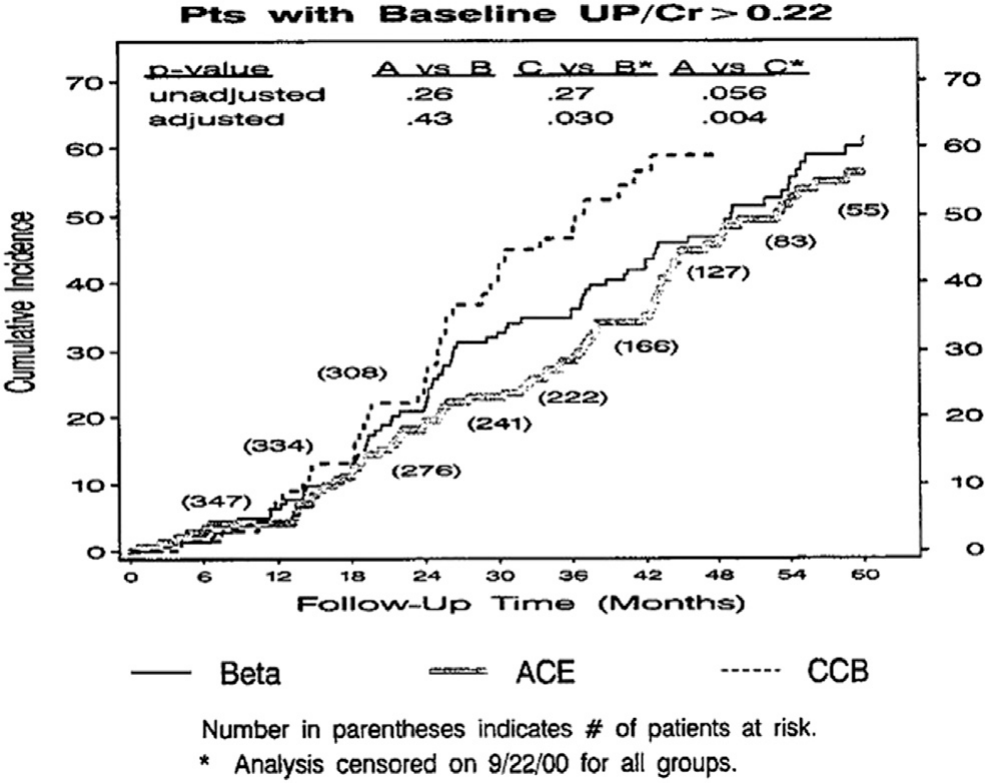
AASK clinical composite events–proteinuria.

Closer inspection of the data highlights the current dilemma: although there is a 30−60% decrease in the number of events with ACEi still a residual event rate of >6%/yr in the trial as a whole and >11%/yr in subjects with urine protein/creatinine >0.22, a mild degree of proteinuria of 200−300mg/day (Figures 1 and 2). In addition it can be seen that the event rate is essentially constant throughout the 5 years of the trial, indicating that remaining patients are still at risk to progress. This finding is similar to that of other trials such as MDRD (Klahr et al., 1994; Hebert et al., 1997), the Collaborative Study Group Trial (Lewis et al., 1993), RENAAL (Brenner et al., 2001) and IDNT (Lewis et al., 2001).

**FIGURE 3:**
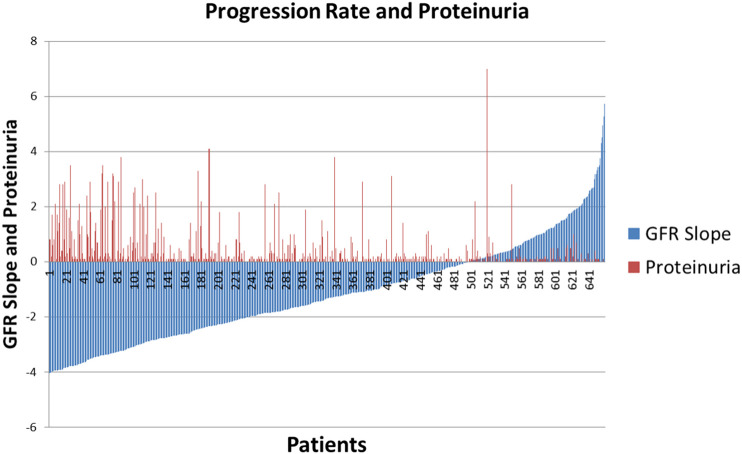
AASK Patients stratified by GFR slope with degree of proteinuria superimposed.

Figure 3 indicates the significant heterogeneity of progression rate of renal disease in the AASK Trial, where the rate of decline of GFR after 6 months in the trial (chronic GFR slope) is depicted in blue for each patient from most rapid decline (negative slope) on the left, to the least rapid decline (positive slope) on the right. The expected rate of decline of GFR with aging is generally assumed to be −1ml/min/yr (Berg, 2006; Murussi et al., 2006), although longitudinal studies have raised questions about this assumption (Lindeman et al., 1985; Lindeman, 1990). Based on this estimate, approximately 30% of the AASK patients in Figure 3 did not progress (right side, slope >−1ml/min/yr) while approximately 30% progressed rapidly (left side, slope <−3ml/min/yr). The figure also shows that proteinuria, the strongest predictor of progression rate reported in literature, is not an ideal predictor in that there are a number of slow progressors with significant proteinuria (red spikes, right), while a significant number of rapid progressors had no or minimal proteinuria (absence of red bars, left) (Subasi et al., 2017). This data is supported by the observation in genetics studies that proteinuria and progression of renal disease may be disparate phenotypes (Fogarty et al., 2000; Krolewski et al., 2006).

### 2.1 Pre-processing of AASK Data to Predict Progression of Renal Disease

**FIGURE 4:**
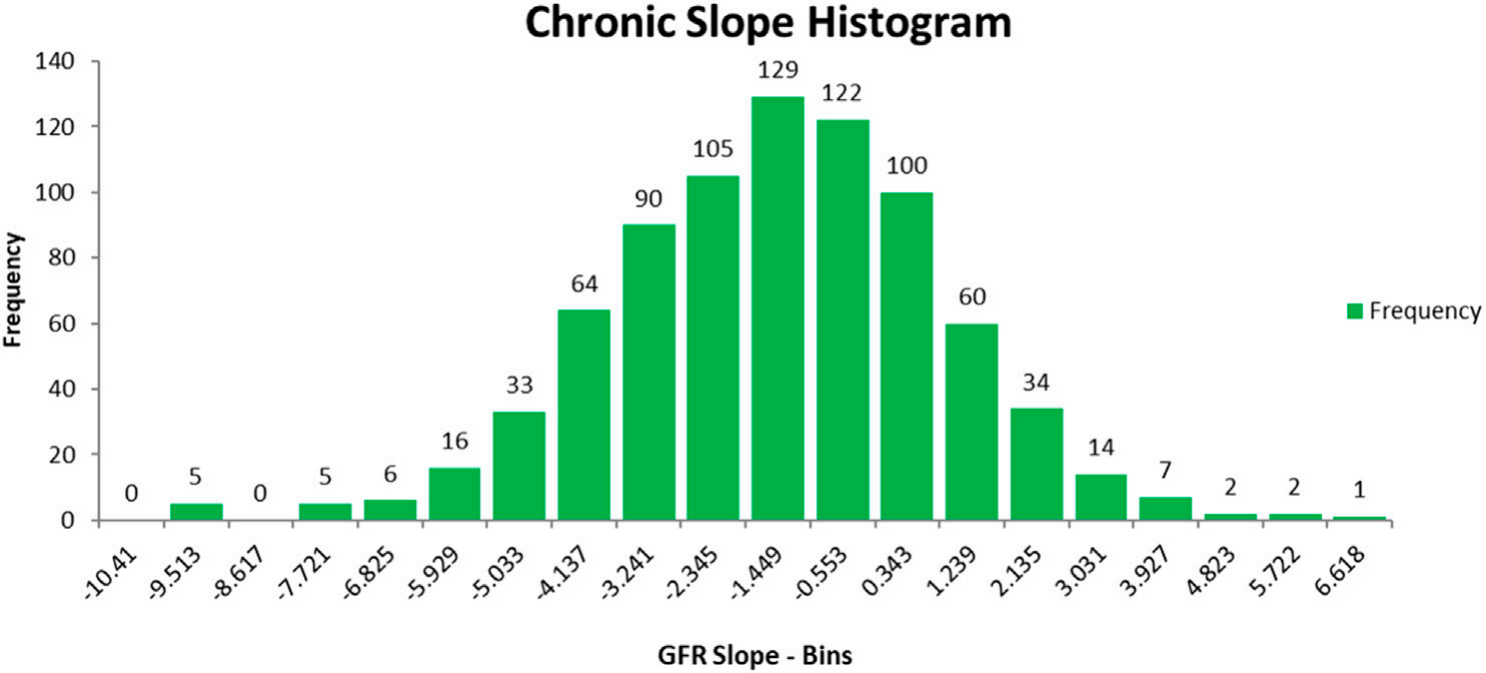
Chronic GFR slope of AASK patients in the reduced data.

An avenue that has not been carefully explored is a data mining approach to detect the combinations of clinical features and/or single nucleotide polymorphisms (SNPs) that better determine the population at risk for progression of CKD. The goal of this section is to identify combinatorial patterns of clinical features and SNPs that can accurately predict progression of the renal disease among AASK patients. In order to achieve this, we perform a study on a selected subset of subjects from the AASK Clinical Trial based on the glomerular filtration slope (GFR) of all AASK patients presented in Figure 3. The original AASK data contains 1,094 African-American patients with 88 clinical features and 130 SNPs. Before we start our analysis, we remove features with more than 80% missing values in the dataset. We then remove AASK patients with missing GFR values and more than 10% missing values. This results in 800 AASK patients with 77 clinical features and 113 SNPs. In order to develop a classification model that can predict the rate of decline of kidney function, we identify two “extreme” groups of patients whose disease progression is “slow” (GFR chronic slope >1ml/min/yr) or “rapid” (GFR chronic slope <−4ml/min/yr). The two subsets of patients, referred to as slow progressors and rapid progressors are selected from the AASK study based on the chronic GFR slope histogram presented in Figure 4. The resulting reduced dataset contains 138 AASK patients identified as rapid progressors and 75 AASK patients as slow progressors.

**FIGURE 5:**
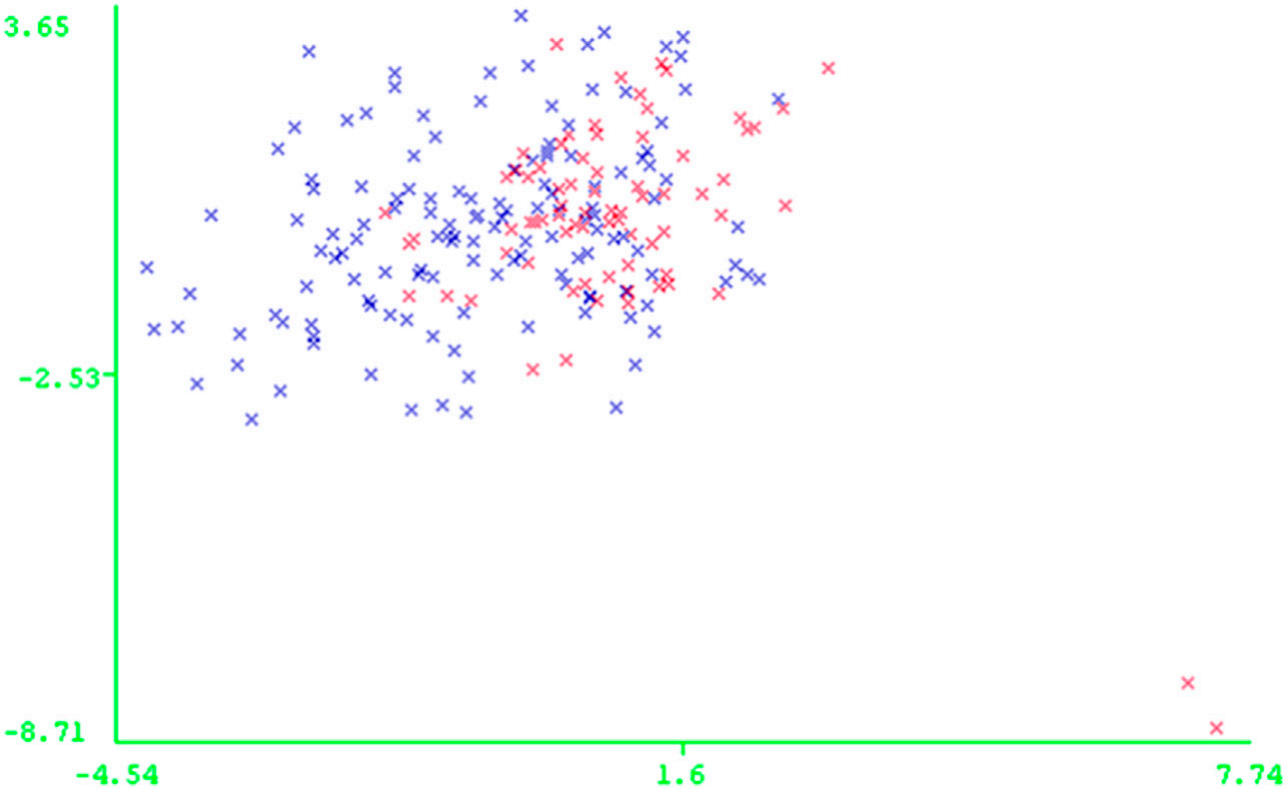
PCA plot of AASK patients in the reduced data: * Rapid Progressors and * Slow Progressors

Figure 5 shows the PCA plot of the AASK patients in the reduced dataset. Table 2 describes the patient population for this study. As can be seen from the table, proteinuria is very different between the two groups of disease progression, which supports the previous studies showing that proteinuria is the strongest predictor of GFR slope progression in AASK (Wang et al., 2006).

**TABLE 2:** Baseline characteristics of study population.

Basic Clinical Features	Rapid Progressors	Slow Progressors
Chronic slope	−5.41±1.36	2.11±1.03
GFR	42.83±13.25	52.30±10.55
Proteinuria	1.12±1.40	0.13±0.20
Age	50.22±11.94	52.52±9.52
Weight (kg)	96.42±22.42	87.52±19.65
(cm)	171.69±10.56	169.21±10.80
BMI	32.69±7.06	30.57±6.09

### 2.2 Identification of Significant Clinical and Genomic Features

The resulting AASK dataset consisting of 138 rapid progressors, 75 slow progressors, 77 clinical features, and 113 SNPs, is further investigated to remove any features irrelevant for the recognition of a rapid progressor as opposed to a slow progressor. In order to obtain a classification model effectively and efficiently, we first apply a correlation-based feature selection procedure (Hall and Smith, 1998) to retain only those relevant features successfully distinguishing between rapid progressors and slow progressors in AASK data. Correlation-based feature selection method evaluates the worth of a subset of features by considering the individual predictive ability of each feature along with the degree of redundancy between them. Subsets of features that are highly correlated with the outcome (rapid/slow progression) while having low intercorrelation are preferred. AASK data is randomly partitioned into ten approximately equal parts; one of these subsets is designated as “test set”, correlation based feature selection is built on the remaining nine subsets which form the “training dataset”, and then evaluated on the cases in the test set. This procedure is repeated ten times, always taking another one of the ten parts in the role of the test set (re-randomizing the patients into ten new subsets and repeating the procedure nine additional times for a total of 100 tests).

Table 3 shows the features selected from ten times 10-folding cross-validation of the correlation-based feature subset selection procedure in WEKA, a commonly used open source data mining software (Hall et al., 2009). The rationale for using small numbers of features is both for ease in collecting the relevant data for prediction on patients from different sources (health systems) and the possibility that finding a small number of novel predictors may help inform studies into the mechanisms and treatment of CKD progression if they suggest new and unexplored pathways. The SNPs and the fact that the alpha-2 agonist antihypertensive medicine use are predictors may help in this manner.

**TABLE 3:** Feature Selection - 10 fold stratified cross validation.

% Absolute Frequency	Feature
90%	α-agonist
100%	Proteinuria
100%	U.Protein/U.Creatinine
70%	GFR value at G1 visit
100%	CHGB-1
90%	PLCG2 rs4399527

## 3 PATTERN-BASED Classification Model to Predict Progression of Renal Disease

### 3.1 Identification of Combinatorial Patterns of Significant Clinical Features and SNPs

Study Subjects analysis provides us with a reduced AASK data, containing 138 rapid progressors and 75 slow progressor with.

• four clinical features: α*-agonist (peripherol base), proteinuria, urine-protein/urine-creatinine, GFR value at G1 visit*, where α-agonist represents the use of peripheral alpha-2 agonist blood pressure medication
• two SNPs: CHGB-1, PLCG2 rs4399527.

These six features were validated using 10 × 10-folding cross-validation experiments on seven commonly used and well-known classification methods, including Random Forest, Decision Trees, Nearest Neighbor, Support Vector Machines, Neural Networks, Logistic Regression, and Naïve Bayes (Hall et al., 2009). In this step the AASK data is randomly partitioned into ten approximately equal parts; one of these subsets is designated as “test set”, a model is built on the remaining nine subsets which form the “training dataset”, and then tested by predicting the classes of patients in the test set using a classification method. This procedure is repeated 10 times, always taking another one of the ten parts in the role of the test set (re-randomizing the patients into 10 new subsets and repeat the procedure nine additional times) for a total of 100 tests for each of the seven classification methods. Table 4 shows average accuracy, sensitivity (proportion of correctly classified rapid progressors), specificity (proportion of correctly classified slow progressors) as well as average precision, recall, F-measure, and area under Receiver Operating Characteristic (ROC) curve.

**TABLE 4:** Cross-validation of classification methods for AASK samples.

Classification Method	Accuracy	Sensitivity	Specificity	Precision	Recall	F-Measure	ROC Area
Random forest	78.33%	83.63%	68.79%	0.71	0.69	0.68	0.86
C4.5 decision tree	76.77%	80.53%	70.18%	0.68	0.70	0.67	0.78
Nearest neighbor	70.21%	76.97%	58.02%	0.59	0.58	0.57	0.68
Support vector machines	72.70%	77.91%	63.34%	0.62	0.63	0.61	0.71
Neural networks	73.07%	78.19%	63.79%	0.63	0.64	0.62	0.81
Logistic regression	75.88%	81.70%	65.39%	0.68	0.65	0.65	0.85
Naïve bayes	70.20%	57.90%	93.02%	0.56	0.93	0.69	0.85

**FIGURE 6:**
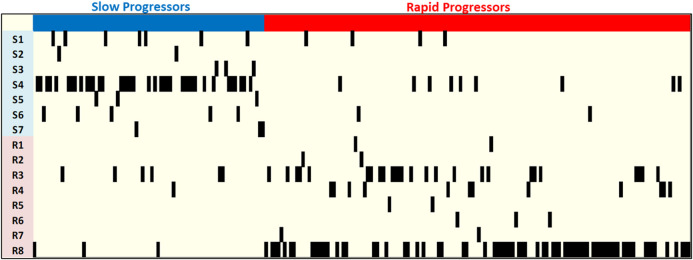
C4.5 decision tree for AASK samples.

As can be seen in Table 4, while Random Forest provides us with highest accuracy, C4.5 Decision Tree (Quinlan, 1993), a non-parametric supervised learning method used for classification and regression, provides the best sensitivity and specificity, i.e., the best prediction for rapid and slow prediction. C4.5 classification model consisting of seven patterns, S1-S7, for slow progressors and eight patterns, R1-R8, for rapid progressors is presented in Table 5 as combinatorial patterns of clinical features and SNPs associated with slow and rapid progression in the AASK dataset. Figures 6 and 8 show the C4.5 decision tree and heatmap corresponding to the combinatorial patterns presented in Table 5, respectively.

**TABLE 5:** C4.5 classification model for AASK samples.

Patterns	C4.5 Classification Model for Renal disease Progression
S1	U. Protein ≤0 and PLCG2 rs4399527=*GC* and CHGB 1*=TT*
S2	U. Protein ≤0 and PLCG2 rs4399527=*GC* and CHGB 1=*CT* and α-agonist ≤0 and Pro./Creat.Ratio > 0.01706
S3	U. Protein ≤0 and PLCG2 rs4399527=*GC* and CHGB 1=*CC*
S4	U. Protein ≤0.5 and PLCG2 rs4399527*=CC* and Pro./Creat.Ratio ≤ 0.15714
S5	U. Protein ≤0.5 and PLCG2 rs4399527=*GG* and CHGB 1*=TT* and 41.4< GFR G1 ≤ 59.5816
S6	U. Protein ≤0.5 and PLCG2 rs4399527=*GG* and CHGB 1=*CT* and Pro./Creat.Ratio > 0.02177
S7	U. Protein ≤0.5 and PLCG2 rs4399527=*GG* and CHGB 1=*CC*
R1	U. Protein ≤0 and PLCG2 rs4399527=*GC* and CHGB 1=*CT* and α-agonist ≤0 and Pro./Creat.Ratio ≤ 0.01706
R2	U. Protein ≤0 and PLCG2 rs4399527=*GC* and CHGB 1=*CT* and α-agonist >0
R3	0 ＜ U. Protein ≤0.5 and PLCG2 rs4399527=*GC*
R4	U. Protein ≤0.5 and PLCG2 rs4399527*=CC* and Pro./Creat.Ratio > 0.15714
R5	U. Protein ≤0.5 and PLCG2 rs4399527=*GG* and CHGB 1*=TT* and GFR G1 ≤ 41.4
R6	U. Protein ≤0.5 and PLCG2 rs4399527=*GG* and CHGB 1*=TT* and GFR G1 > 59.5816
R7	U. Protein ≤0.5 and PLCG2 rs4399527=*GG* and CHGB 1=*CT* and Pro./Creat.Ratio ≤ 0.02177
R8	U. Protein > 0.5

**FIGURE 7:**
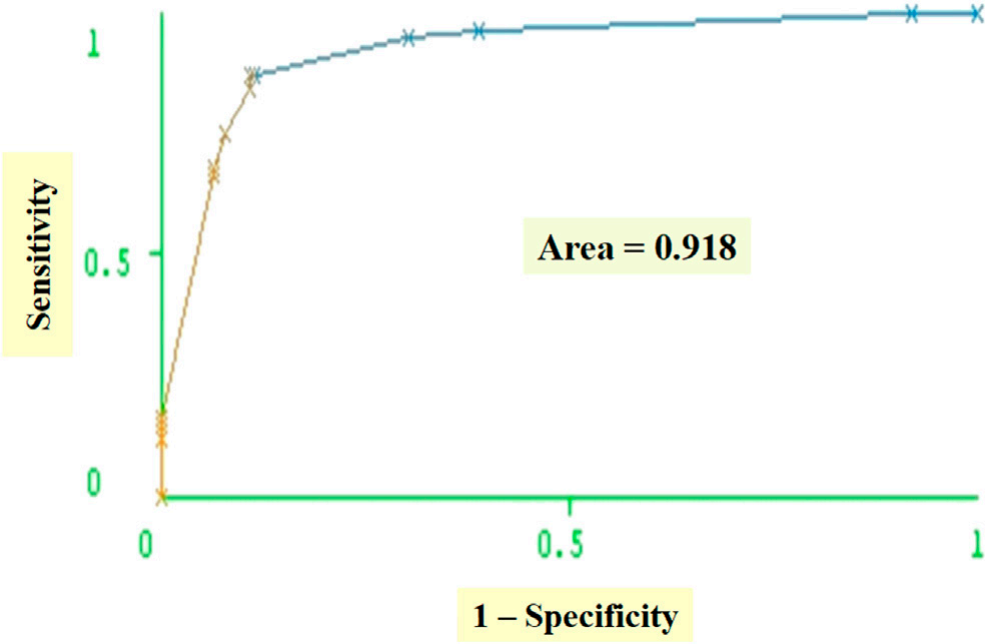
Heatmap of the C4.5 patterns for AASK samples.

**FIGURE 8:**
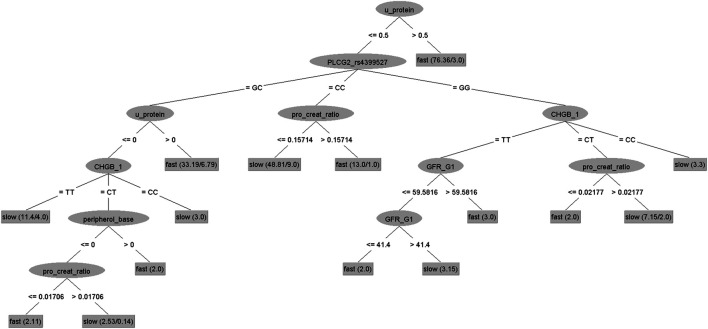
Receiver operating curves (ROC).

### 

 The pattern characteristics including

• *rapid prevalence*: proportion of rapid progressors covered by a pattern to the total number of rapid progressors,
• *slow prevalence*: proportion of slow progressors covered by a pattern to the total number of slow progressors,
• *rapid homogeneity*: proportion of rapid progressors covered by the pattern,
• *slow homogeneity*: proportion of slow progressors covered by the pattern,
• *degree*: number of conditions appear in the description of the pattern of the C4.5 classification model are given in Table 6.

**TABLE 6:** C4.5 decision tree pattern characteristics.

Pattern	Homogeneity (%)	Slow prevalence	Rapid prevalence	Degree
S1	63.64%	9.33%	5.33%	3
S2	100%	2.67%	0%	5
S3	100%	4%	0%	3
S4	80.85%	50.67%	12%	3
S5	100%	4%	0%	4
S6	71.43%	6.67%	2.67%	4
S7	100%	4%	0%	3
R1	100%	1.45%	0%	5
R2	100	1.45%	0%	4
R3	81.25%	18.84%	4.35%	2
R4	92.31%	8.70%	0.72%	3
R5	100%	1.45%	0%	4
R6	100%	2.17%	0%	4
R7	100%	1.45%	0%	4
R8	96.10%	53.62%	2.17%	1

### 3.2 Validation of Combinatorial Patterns

We remark that the C4.5 classification model given in Table 5 consists of explicit patterns, where the four clinical features and two SNPs selected in Identification of Significant Clinical and Genomic Features are assigned threshold values. Note that patterns S1-S7 exhibit high homogeneity for the slow progressors and R1-R8 exhibit high homogeneity for the rapid progressors in AASK data. For example, patterns S2, S3, S5, S7 have 100% homogeneity, meaning that all patients covered by each of these patterns are slow progressors. Similarly, the homogeneity of patterns R1, R2, R5, R6, R7 is also 100%, i.e., all patients covered by each of these patterns are rapid progressors. We refer to such patterns as pure patterns associated with the respective subgroups of AASK patients. We also remark that the classification model contains fuzzy patterns, S1, S4, S6, R3, R4, R8, i.e., patterns with homogeneity <100%. For example, the homogeneity of pattern S4 is 81%, meaning that 81% of the patients covered by pattern S4 are slow progressors and the remaining 19% of the patients covered by this pattern are rapid progressors in AASK Clinical Trial.

As for the prevalence, patterns S4 and R8 are significant patterns, S4 covering 51% of all slow progressors, but only 12% of the rapid progressors and R8 covering 54% of all rapid progressors, but only 2% of the slow progressors in the data. While the other patterns in the classification model does not exhibit high prevalence in the associated subgroups within the data, they are still required to predict the progression of all AASK patients in the study. Finally, we observe that these patterns use small number of features of AASK patients. The degrees of the patterns (number of features used in pattern description) range from one to 5. Note that according to pattern R8, the U. Protein levels of 54% of rapid progressors exceeds 0.5 and 96% of the patients covered by this pattern are rapid progressors. Similar observations can be done for other patterns forming the classification model in Table 5.

Based on the 10 × 10-folding cross-validation experiments, the classification model correctly classifies 80.53% of rapid progressors and 70.18% of slow progressors and exhibits an average accuracy of 76.77% with 0.68 precisiom, 0.70 recall, and 0.67 F-measure, validating the distinguishing power of the classification model for the AASK patients in our study. As another measure of the effectiveness of the classification model at predicting rapid or slow progressors, we generate receiver operating characteristic (ROC) curve that shows how much the classification model is capable of distinguishing between the rapid progressors and slow progressors in AASK Clinical Trial. ROC curve is obtained by plotting sensitivity (true positive rate) against 1−specificity (false positive rate). Based on 10 × 10-folding cross-validation experiments, the area under the ROC curve is 0.78. ROC curve corresponding to the C4.5 classification model (built on entire dataset) in Table 5 is shown in Figure 8.

Thus, we can conclude that the combinatorial patterns forming the classification model in Table 5 are high quality decision rules that can be easily interpreted by medical experts, allowing them to target the clinical features and SNPs associated with the progression of the renal disease to develop new therapies.

## Data Availability Statement

The datasets generated for this study can be found in the African American Study of Kidney Disease and Hypertension Study (Clinical Trial) (AASK Trial) https://repository.niddk.nih.gov/studies/aask-trial/.

## Author Contributions

ES, ML, and MMS are senior co-authors who designed and supervised the entire project and participated in writing the manuscript. MMM, TB, and MSM participated in the study design and performed the combinatorial analysis and participated in writing the manuscript. KC, EC, ZA, and RP were involved in various steps of the combinatorial analysis.

## Funding

ML, ES, and MMS’s work was supported by National Institutes of Health—Grant number: 5R21DK67468. KC and EC’s work was supported by National Science Foundation (NSF) Research Experience for Undergraduates (REU) Grant—Award number: 1,359,341.

## Conflict of Interest

The authors declare that the research was conducted in the absence of any commercial or financial relationships that could be construed as a potential conflict of interest.

## Footnotes

1 Chronic Kidney disease Surveillance Project, Center for disease Control and Prevention—http://nccd.cdc.gov/ckd/
